# A rare case of MRI-induced thermal burn: Clinical implications and safety awareness

**DOI:** 10.1016/j.radcr.2025.01.069

**Published:** 2025-03-08

**Authors:** Amer Abu-Shanab, Hamzeh Nasr, Ahmed Mohd, Mahmoud Shaweish, Ahmad Abdulraheem, Doantrang Du

**Affiliations:** a Department of Internal Medicine, Rutgers Health-Monmouth Medical Center, Long Branch, NJ, USA; b Faculty of Medicine, The Hashemite University, Zarqa, Jordan; c Department of Internal Medicine, Medstar Georgetown Washington Hospital Center, DC, USA

**Keywords:** MRI-induced burns, Radiofrequency energy, Thermal injury, Patient safety, Case report

## Abstract

Magnetic Resonance Imaging (MRI) is a widely used, noninvasive diagnostic tool in clinical practice, with millions of scans performed annually. Despite its safety profile, MRI is associated with several potential side effects, including rare but serious complications such as thermal burns. These burns typically result from the interaction of radiofrequency (RF) energy with conductive materials in contact with the skin, or from improper positioning during the scan. This case report details a 76-year-old female who developed a thermal burn on her left elbow after undergoing an MRI of her right shoulder. The patient initially experienced localized heat, redness, and mild tenderness at the site, which later developed into an open wound, leading to a diagnosis of cellulitis. However, further evaluation revealed the wound to be a stage III burn, likely resulting from RF energy exposure during the MRI procedure. The patient had a history of diabetes, a condition that may have contributed to impaired thermoregulation, increasing her risk for thermal injury. The burn was managed with wound debridement, antibiotics, and regular follow-up care, ultimately healing over an 8-week period. This case underscores the importance of understanding the mechanisms of MRI-induced burns, the need for stringent safety protocols, and the role of postprocedure patient education. It also highlights the potential for delayed diagnosis of MRI-related burns, which can be mistaken for other conditions such as cellulitis. Early recognition and appropriate management are critical to preventing complications and ensuring optimal patient outcomes.

## Background/Introduction

Magnetic Resonance Imaging (MRI) has emerged as a valuable diagnostic tool in clinical applications since the mid-1980′s. MRI is generally a safe, noninvasive diagnostic technique with nearly 40 million scans carried out annually in the United States [1]. However, like any medical procedure, it is not without potential side effects. Common side effects of MRI include claustrophobia [2], noise-induced discomfort [3], and dizziness or nausea [1]. More serious but rare side effects include allergic reactions to contrast agents and nephrogenic systemic fibrosis in patients with severe kidney dysfunction [4,5].

Thermal burns, although rare, represent a potentially serious complication of MRI [6]. Of MRI adverse effects, 59% that are reported to the Food and Drug Administration (FDA) include thermal injuries [7]. The exact prevalence of MRI-induced burns is difficult to determine due to underreporting [7]. These burns typically result from the interaction between radiofrequency (RF) energy and conductive materials in contact with the patient's skin [8]. This case report presents a rare instance of an MRI-induced skin burn, highlighting the importance of adherence to safety protocols and the need for increased awareness among healthcare professionals and patients about this uncommon but potentially serious complication of MRI procedures.

## Case presentation

This case presents a 76-year-old female patient, with a past medical history of diabetes malleus type 2, hypertension, hypothyroidism, and migraine disorder, who presented to the emergency department (ED) for redness and pain at the left elbow. Two months prior to her presentation, the patient fell down from her bed injuring her right shoulder. Since the incident, she had pain in her shoulder for which she had an X-ray which was unraveling. The patient was unable to raise her right arm above 90 degree that was associated with pain with passive range of motion at the right shoulder. The empty can test was positive and rotator cuff tear was suspected for which MRI of the right shoulder was ordered.

**Fig. 1:**
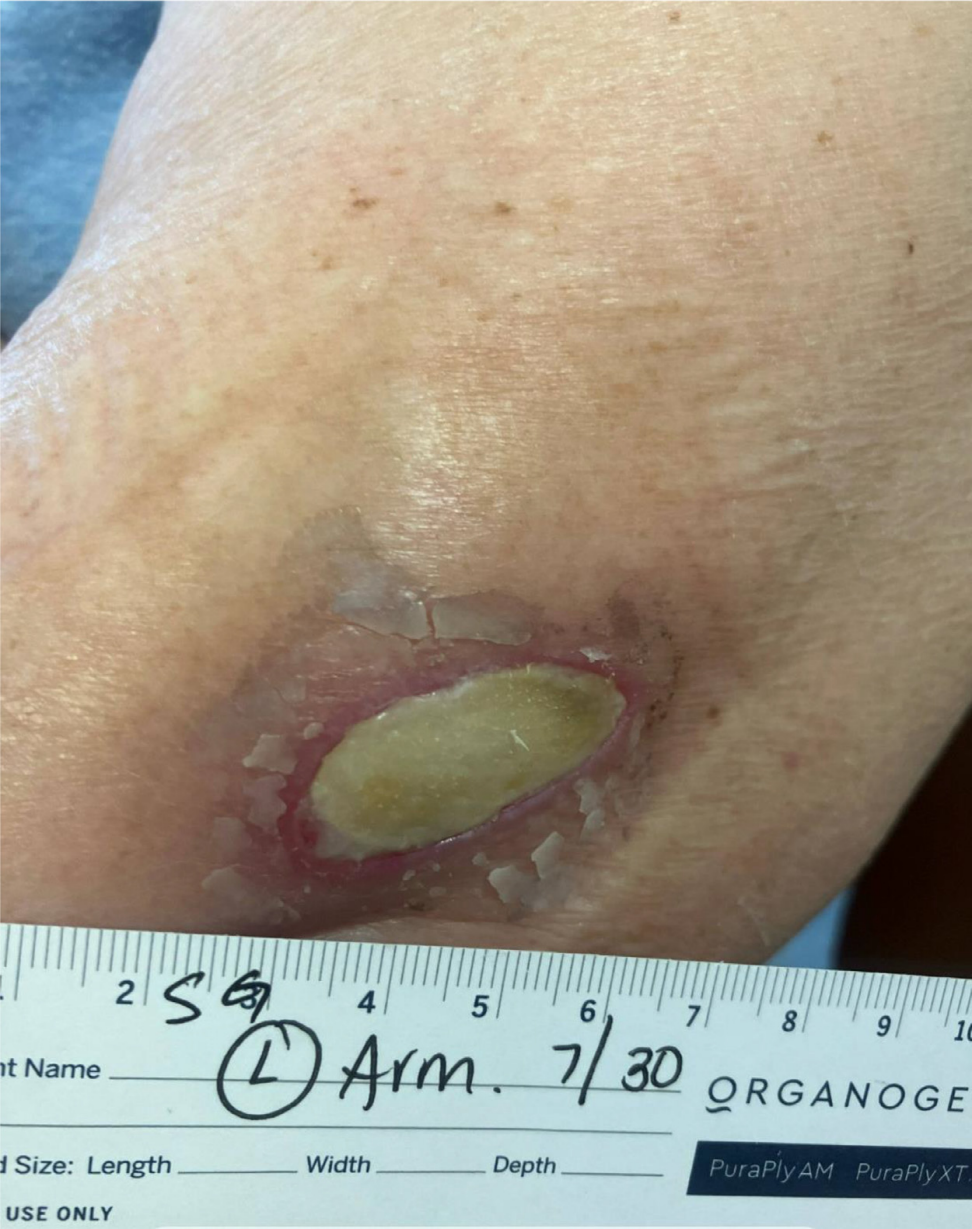
Left elbow thermal burn lesion on presentation.

**Fig. 2:**
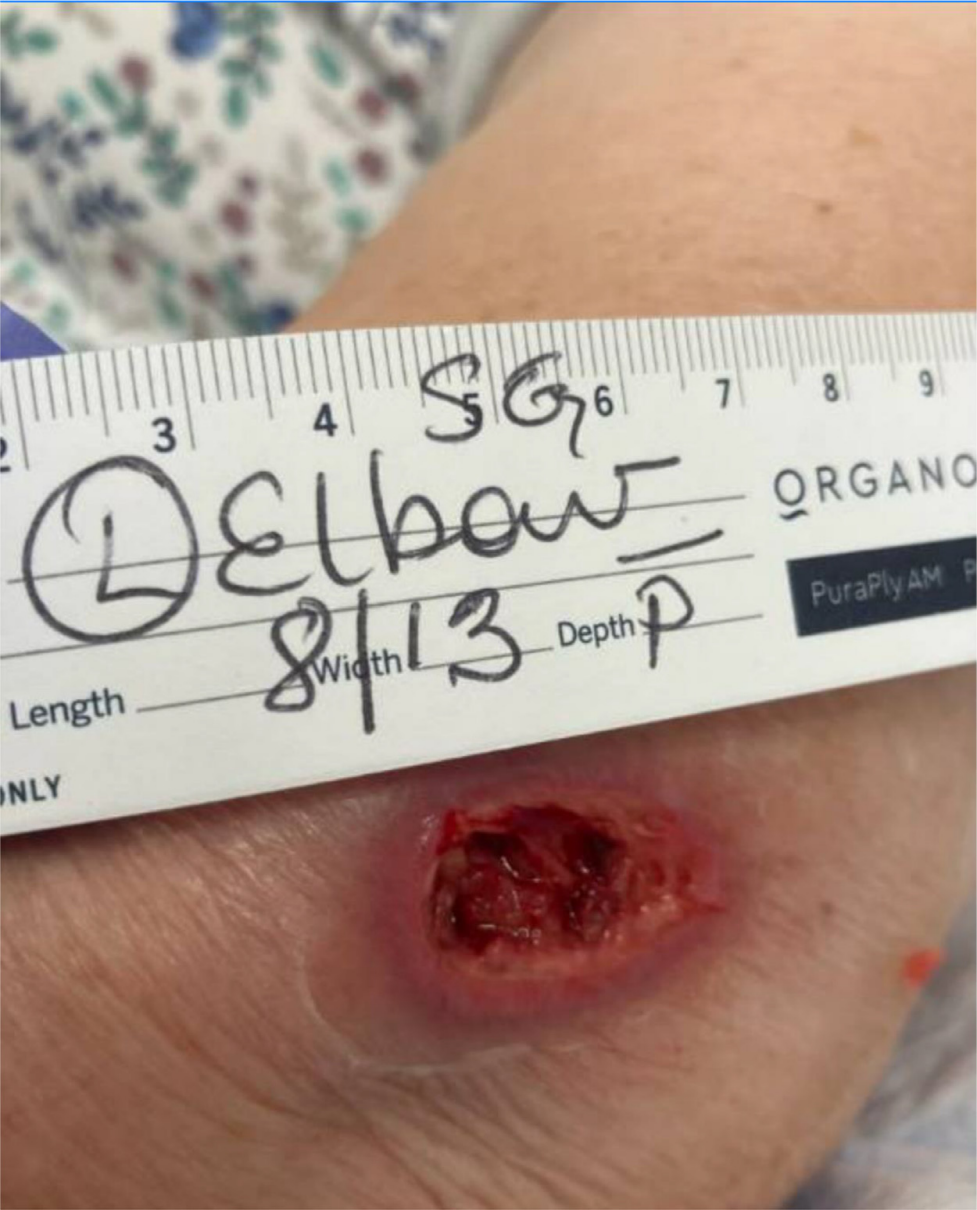
Left elbow thermal burn lesion after debridement and 2 weeks of antibiotics.

**Fig. 3:**
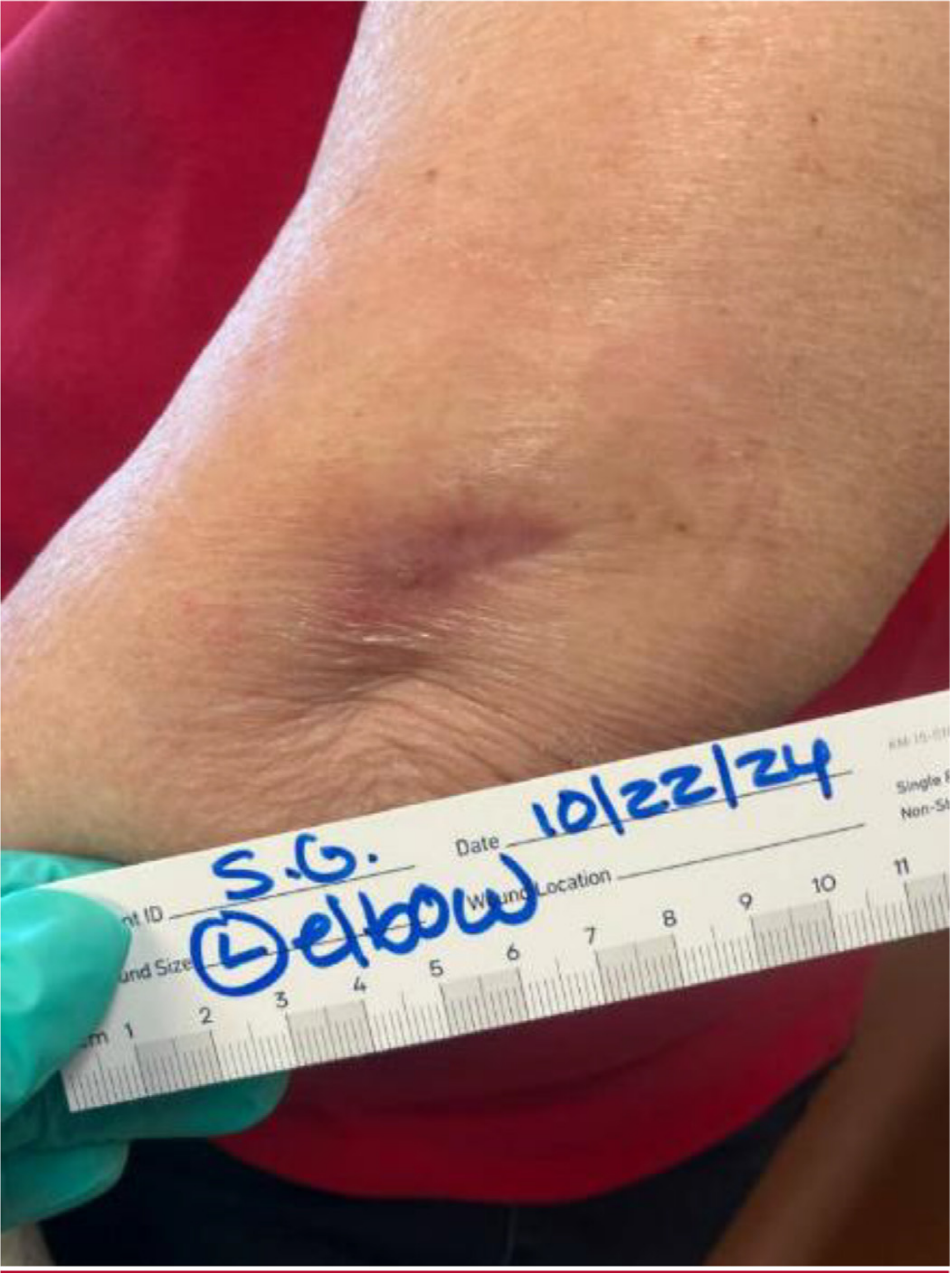
Left elbow thermal burn lesion after debridement and 2 months of antibiotics.

One week prior to today's presentation she underwent an MRI for the right shoulder. During the imaging, she felt heat at her left elbow and right after the imaging, she noticed redness and mild tenderness at the same site. She ignored it initially hoping that it will go away. Later, she presented to the ED with an open wound at the elbow with worsening in the redness around it. During examination, an elliptical shaped scab to the left elbow with moderate surrounding erythema, without purulence or discharge was noted. Figures 1, 2, and 3 demonstrate the lesion found on the left elbow of the patient. She denied fever, chills, rigors, and fatigue. She was diagnosed with cellulitis and discharged from the ED on cephalexin, with a referral to the wound care clinic.

She took her medication as prescribed and presented to the wound clinic for follow up. On follow up, she had a nonhealing 4 × 2 cm wound with a thick scab. The lesion was red and swollen but not tender. There was no fluctuance nor discharge. She was diagnosed with stage III burns with thick eschar. The X-ray of the left elbow was normal. The wound was debrided, and she was started on Bactrim and topical gentamicin with close follow ups. Sterile dressing was done on a weekly base by the wound care team. The burn was improving over time and was in a healing phase after 8 weeks of dressing and topical antibiotics.

## Discussion

This case presents a 76-year-old female patient experiencing a thermal burn post-MRI imaging. This incident highlights the importance of understanding the mechanisms by which MRI can potentially cause skin burns and the need for stringent safety protocols.

MRI scanners employ powerful radiofrequency (RF) pulses to create images, which causes RF heating. Particularly in areas where a concentrated electromagnetic field is applied, these radiofrequency waves have the potential to heat tissue [8]. It's possible that the patient's left elbow was placed in an area with a strong radiofrequency field in this instance.

Another explanation might be conductive loop formation, where the body part might be positioned in a way that created a conductive loop (e.g., skin-to-skin contact) [6]. A case study published in 2017 by Mandel and her colleagues [6] presented a 48-year-old man who experienced a second degree burn on his thighs bilaterally following an MRI. It was believed that the patient mistakenly pushed his thighs together while inside the scanner, creating a closed loop conduction circuit that led to a thermal burn. The significance of proper posture during MRI is demonstrated by the case.

In addition, the presence of undetectable metallics in clothing could be a possible source for thermal burn. Previous case studies showed that nondetectable metallic material, which is found in popular athletic clothing presented a serious risk for MRI cutaneous burns. The metallic fiber in the clothing is difficult to detect by standard MRI screening methods [9,10]. In this case study [10], an MRI was performed on a 40-year-old woman to evaluate her thigh muscles. Following the MRI, both her thighs showed linear redness and swelling that matched the vertical lines on the jogging clothes she was wearing. The redness eventually turned into blistering eruptions a week later. The jogging trousers' material list mentioned 100% polyester and did not include any mention of metal. They later found that the vertical lines of the jogging pants might have been made of light, thin metal fibers after contacting the company's customer service.

Impaired thermoregulation in elderly, particularly those with diabetes, may have contributed to reduced peripheral sensation and impaired thermoregulation [11]. These factors increased the possibility of thermal injury in our patient. Finally, despite our patient denying that, but the application of a cream or a lotion that contains heavy material might increase the absorption of the RF, thus increase the temperature of the area.

The delayed presentation and initial misdiagnosis as cellulitis underscore the importance of post-MRI patient education and follow-up. Patients should be instructed to report any unusual sensations or skin changes promptly after an MRI procedure.

## Conclusion

This case emphasizes the need for increased awareness among healthcare providers about the potential for MRI-induced burns, even in the absence of obvious risk factors. It also highlights the importance of prompt recognition and appropriate management of such injuries to prevent complications and ensure optimal patient outcomes.

## Patient consent

The patient provided written informed consent to publish the information of her post-MRI burn and any accompanying images.
